# A *Drosophila* Model of ALS: Human ALS-Associated Mutation in VAP33A Suggests a Dominant Negative Mechanism

**DOI:** 10.1371/journal.pone.0002334

**Published:** 2008-06-04

**Authors:** Anuradha Ratnaparkhi, George M. Lawless, Felix E. Schweizer, Peyman Golshani, George R. Jackson

**Affiliations:** 1 Department of Neurology, David Geffen School of Medicine at the University of California Los Angeles, Los Angeles, California, United States of America; 2 Department of Neurobiology, David Geffen School of Medicine at the University of California Los Angeles, Los Angeles, California, United States of America; 3 Center for Neurobehavioral Genetics, Semel Institute for Neuroscience and Human Behavior, David Geffen School of Medicine at the University of California Los Angeles, Los Angeles, California, United States of America; University of Cambridge, United Kingdom

## Abstract

ALS8 is caused by a dominant mutation in an evolutionarily conserved protein, VAPB (vesicle-associated membrane protein (VAMP)-associated membrane protein B)/ALS8). We have established a fly model of ALS8 using the corresponding mutation in *Drosophila* VAPB (dVAP33A) and examined the effects of this mutation on VAP function using genetic and morphological analyses. By simultaneously assessing the effects of VAP^wt^ and VAP^P58S^ on synaptic morphology and structure, we demonstrate that the phenotypes produced by neuronal expression of VAP^P58S^ resemble VAP loss of function mutants and are opposite those of VAP overexpression, suggesting that VAP^P58S^ may function as a dominant negative. This is brought about by aggregation of VAP^P58S^ and recruitment of wild type VAP into these aggregates. Importantly, we also demonstrate that the ALS8 mutation in dVAP33A interferes with BMP signaling pathways at the neuromuscular junction, identifying a new mechanism underlying pathogenesis of ALS8. Furthermore, we show that mutant dVAP33A can serve as a powerful tool to identify genetic modifiers of VAPB. This new fly model of ALS, with its robust pathological phenotypes, should for the first time allow the power of unbiased screens in *Drosophila* to be applied to study of motor neuron diseases.

## Introduction

Amyotrophic lateral scelerosis (ALS) is a progressive neurodegenerative disease characterized by loss of motor neurons in the spinal cord (“lower” motor neurons), brainstem, and cortex (“upper” motor neurons). The combination of muscular atrophy and fasciculations secondary to lower motor neuron loss, as well as spasticity due to upper motor neuron death, gives rise to the characteristic clinical picture of the disorder [1]. Recently, Zatz and coworkers identified a new locus for ALS/motoneuron disease at 20q13.3 (ALS8) in a large Brazilian family [2]. ALS8 is an autosomal dominant slowly progressive disorder characterized by fasciculation, cramps, and postural tremor. Autonomic abnormalities are also seen [3]. ALS8 is caused by a missense mutation in the vesicle-associated membrane protein (VAMP)/ synaptobrevin-associated membrane protein B gene (VAPB) [2].

VAPB is a type II integral membrane protein comprised of an amino terminal major sperm protein (MSP) domain that bears 22% sequence identity to the major sperm protein, a central coiled-coil domain, and a membrane anchored carboxy terminal domain [4], [5]. This protein is phylogenetically well conserved; homologs of VAPB are found across species from yeast to mammals [2], [6], [7]. Several different functions of VAP are known. A conserved function involves recruitment of FFAT motif containing proteins to the ER [6], [8]. In addition, VAP has a proposed role in vesicle trafficking [9], [10]. In yeast, Scs2, the yeast homolog of VAP, plays a role in the transcriptional regulation of the INO1 gene, which is important for the synthesis of inositol [11]. In *Drosophila*, dVAP33A, the fly homolog of VAPB (hereafter referred to as VAP), binds to the microtubule network and regulates bouton size at the neuromuscular junction [12].

The ALS8-associated missense mutation occurs in the conserved MSP domain of VAPB. The mutation appears to alter the protein's subcellular distribution *in vitro*, with the mutant failing to colocalize with ER or Golgi markers and forming intracytoplasmic aggregates [2]. Subsequent biochemical studies have demonstrated a role for VAPB in the unfolded protein response (UPR); the ALS8 mutation impairs the function of the wild type protein in the UPR [13]. More recently, Hoogenraad and colleagues have demonstrated VAPA and VAPB are reduced in ALS patients and in mutant SOD1 transgenic mice, suggesting a wider role for VAPB in sporadic and familial forms of ALS [14].

Here, we have used *Drosophila* to study the effect of the ALS8-associated mutation using fly VAP. We demonstrate that, unlike the wild type protein, misexpression of the ALS-associated VAP mutation has phenotypic effects reminiscent of loss of function mutations in the endogenous gene. The mutant protein aggregates, is ubiquitinated, recruits wild type protein into aggregates, and interferes with its function, consistent with a dominant negative mechanism. Importantly, we find that expression of the mutant protein interferes with BMP signalling at the neuromuscular junction, which not only suggests a possible regulation of this pathway by VAP, but also could have implications in understanding disease pathogenesis. This model thus provides a novel platform for genetic dissection of motor neuron death that may provide powerful insights into both sporadic and familial ALS.

## Results

### Expression of VAP^P58S^ Impairs VAP Activity *In Vivo*

In order to understand how the ALS8-associated mutation affects VAP function and other cellular processes, we used a gain of function approach. We generated transgenic flies carrying the wild type (VAP^wt^) and mutant (VAP^P58S^) forms of VAP using the GAL4-responsive UAS expression construct [15]. As a first step, we expressed both constructs in a variety of tissues using different GAL4 driver lines and examined the effect of each on viability of the organism. These included neuronal (*elav*), glial (*repo*), muscle/mesoderm (24B), and ubiquitous drivers (tubulin and actin; Table 1). Overexpression of VAP^wt^ in muscle using 24B-GAL4 [15] was lethal during early larval life (first or second instar). When expression was carried out using a more restricted GAL4 line, G14-GAL4 [16], the animals were able to survive through pupal development but were unable to eclose from their pupal cases and died as pharate adults. In contrast to wild type, overexpression of VAP^P58S^ in muscle was not lethal; viable adult flies were obtained at 25°C (Table 1). However, at 30°C, expression of mutant VAP in muscle was lethal; the animals died during the pupal stage (Table 1). Surprisingly, neuronal expression of neither VAP^wt^ nor VAP^P58S^ had any effect on viability at either temperature.

**Table 1:** A comparison of the effects of overexpression of VAP^wt^ and VAP^P58S^ in various tissues X, lethality.

	*elav*-GAL4		24B-GAL4		tub-GAL4		act-GAL4		repo-GAL4	
	25°	30°	25°	30°	25°	30°	25°	30°	25°	30°
VAP^wt^	√	√	×	×	×	×	×	ND	√	ND
VAP^P58S^	√	√	√	×	√	×	√	ND	√	ND

Check, viable. ND, not done.

To ensure that their differential effects were not due to differences in the expression level of VAP^wt^ and VAP^P58S^ transgenes, we performed an immunoblot analysis. Both transgenes were found to have comparable levels of protein expression (Figure S1). These data suggest that the ALS8 mutation impairs the biological activity of VAP such that higher expression of the mutant protein is required to bring about effects similar to those elicited by the wild type protein.

**Figure 1:**
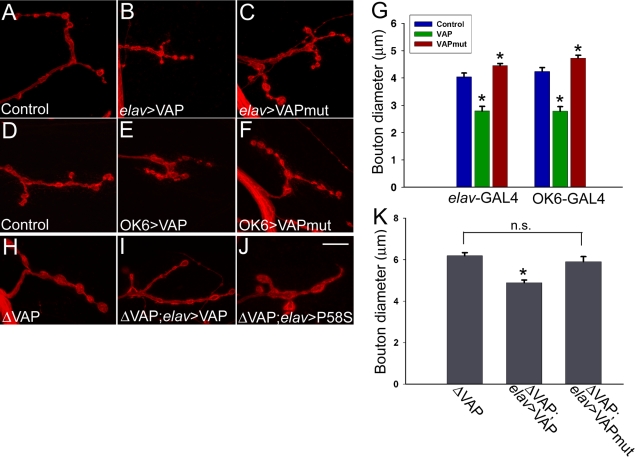
VAP^wt^ and VAP^P58S^ (VAPmut) have differential effects on synaptic morphology at the neuromuscular junction. Shown are representative synapses on muscle 4 of 3^rd^ instar larvae stained with anti-HRP, which labels the presynaptic membrane. The effect of neuronal overexpression of VAP^wt^ and VAP^P58S^ on bouton morphology was assessed using the pan-neural *elav-*GAL4 driver (A–C) and the more restricted OK6-GAL4 (D–F). (H–J) Effect of neuronal expression of wild type and mutant VAP on bouton size in VAP^Δ166^ animals. (A and D) Control synapses on muscle 4 (*elav-*GAL4/+ and *OK6*-GAL4/+ respectively). Neuronal overexpression of VAP^wt^ results in smaller boutons (B and E), while expression of VAP^P58S^ leads to an increase in bouton size (C and F). (G). Graphical analysis of bouton size effects. Here and throughout, values shown are mean±SEM. For both drivers, the average size of boutons in animals expressing VAP^wt^ and VAP^P58S^ was found to be significantly different not only with respect to one other but also controls (*, *p*<0.001, one way ANOVA with Sidak-Holm multiple comparison test). The bouton sizes measured was as follows (µm): *elav-*GAL4/+ = 4.03±0.14 (n = 16); *elav*-GAL4/VAP^wt = ^2.79±0.17 (n = 16); *elav*-GAL4/ VAP^P58S = ^4.45±0.08 (n = 24); OK6-GAL4/+ = 4.24±0.14 (n = 20); OK6-GAL4/VAP^wt^ = 2.78±0.17 (n = 11); OK6-GAL4/ VAP^P58S^ = 4.72±0.11 (n = 24). (H–J) A representative synapse on muscle 4 is shown in each panel. (H) In VAP^Δ166^ mutants, enlarged boutons are observed. (I) Neuronal expression of VAP^wt^ using *elav*-GAL4 rescues this phenotype by reducing bouton size. (J) Expression of VAP^P58S^ had no effect on bouton size. (K) Histogram comparing bouton size for the genotypes in panels H–K. Mean bouton diameter (µm) for VAP^Δ166^;UAS-VAP^wt^/*elav*-GAL4 (4.89±0.13 (n = 27)) was significantly different from VAP^Δ166^ (6.18±0.15 (n = 24); *p*<0.001, one way ANOVA with Sidak-Holm comparison). However, bouton size in VAP^Δ166^;UAS-VAP^P58S^/*elav*-GAL4 (5.9±0.24 (n = 30) did not differ significantly from VAP^Δ166^ . n.s., not significant. Scale bar, 10 µm.

It has been previously reported that VAP regulates bouton size at the neuromuscular junction in a dose-dependent manner [12]. Increased expression of VAP leads to smaller and more numerous boutons whereas, reduced VAP expression results in fewer but larger boutons. We therefore examined the effect of neuronal overexpression of VAP^P58S^ on bouton morphology at the third instar larval neuromuscular junction. Consistent with the findings of Bellen and coworkers [12], we found that expression of VAP^wt^ using the pan-neuronal *elav*-GAL4 or the more restricted OK6-GAL4 driver [16] produced smaller boutons (Figure 1B and E). In contrast, animals overexpressing VAP^P58S^ had larger boutons (Figure 1C and F).

We measured and compared bouton size of control and animals overexpressing VAP^wt^ and VAP^P58S^ by calculating the average diameter of boutons on muscle 4. Only the largest diameter of each bouton was measured in each case. The average bouton diameter of at least 15 synapses was calculated for each of the genotypes. Pan-neuronal expression of VAP^P58S^ caused a small but significant increase in bouton size as compared to control animals and wild type overexpressors (Figure 1G). Similar results were obtained with the OK6-GAL4 (Figure 1G). As described previously [12], we also observed an increase in bouton number with neuronal expression of wild type VAP. However, overexpression of mutant VAP had no effect on bouton number as compared to control (data not shown). Interestingly, overexpression of VAP in neurons altered the staining pattern of Discs large (DLG), which labels the subsynaptic reticulum (SSR) on the post synaptic surface. Unlike wild type, DLG staining in these animals appeared broad and diffuse (data not shown), indicating that neuronal expression of VAP can influence the organization of post-synaptic elements, consistent with a non cell autonomous action of VAP.

Next, we examined the effects of expression of VAP^P58S^ on bouton size in *Drosophila* mutants that lack full-length endogenous VAP protein. We expressed VAP^wt^ and VAP^P58S^ in the CNS of the VAP^Δ166^ mutant. This mutant is a partial loss- of- function allele of VAP that lacks approximately 100 residues at the amino terminal region of the protein. Hemizygous VAP^Δ166^ males survive until the third instar larval stage, but most of them die as pupae and very few “escaper” males are observed on eclosion [12]. Similar to VAP null mutants, VAP^Δ166^ larvae have enlarged boutons at the larval neuromuscular junction, and this phenotype was rescued by neuronal expression of wild type VAP (Fig 1I). However, we were unable to rescue this phenotype with VAP^P58S^ (Fig 1J). We measured the average bouton size in all three genotypes: the mean bouton diameter in VAP^Δ166^ males was 6.18±0.15 µm (Figure H and K). In the presence of neuronally expressed wild type VAP, however, these boutons were significantly smaller, having an average diameter of 4.89±0.13 µm (Figure 1I and K), similar to the size observed in control animals (Fig 1G, blue bar). In contrast, neuronal expression of VAP^P58S^ in VAP^Δ166^ mutants did not cause any significant change in bouton size (5.90±0.24 µm; Figure 1J and K). Thus, expression of wild type but not mutant VAP was able to rescue the enlarged bouton phenotype in dVAP^Δ166^ mutants.

### VAP^P58S^ May Function as a Dominant Negative

**Figure 2:**
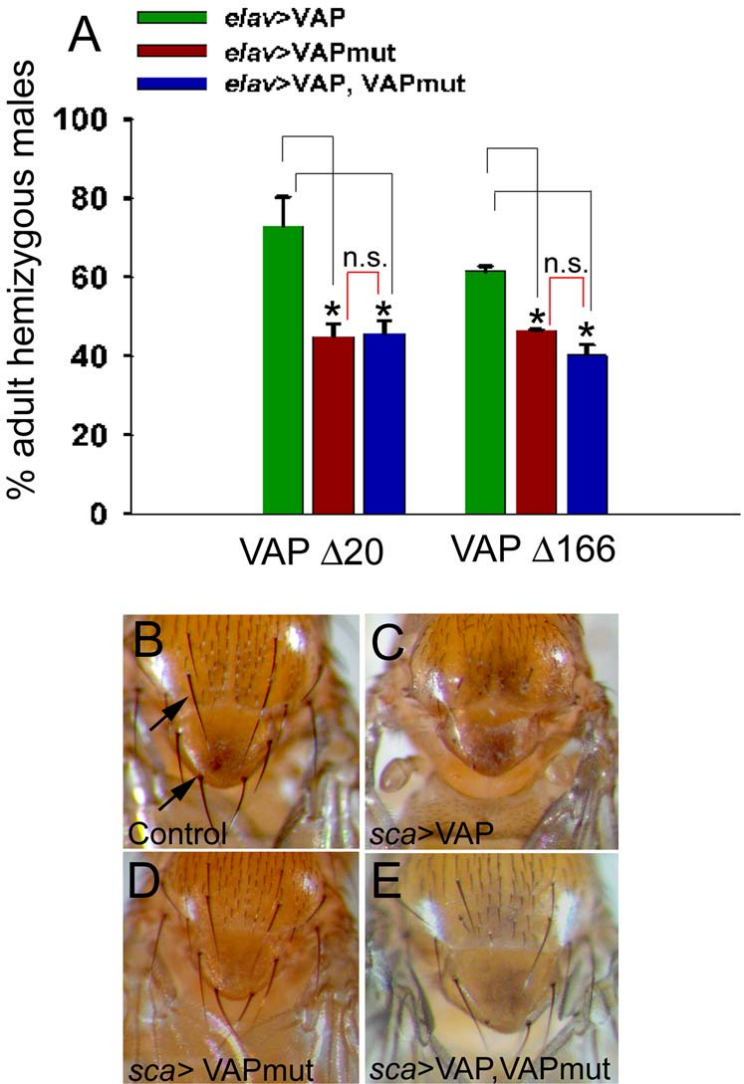
VAP^P58S^ appears to function as a dominant negative by inhibiting the activity of wild type VAP. (A) Histogram comparing the rescue from lethality of VAP^Δ20^ (null mutant) and VAP^Δ166^ (partial deletion), using neuronal expression of VAP^wt^, VAP^P58S^, or a combination of both. The ordinate represents the percentage of mutant hemizygous males obtained (i.e., VAP mutant hemizygous males/VAP mutant hemizygous males+balancer chromosome males). Each bar represents the mean obtained from at least 2–3 independent experiments. Both VAP^P58S^ and VAP^wt^ are able to rescue the larval lethality observed in VAP^Δ20^ and VAP^Δ166^ mutants. However, the percentage of mutant males obtained with VAP^wt^ is higher (72.9±7.3 for VAP^Δ20^ and 61.5±1.2 for VAP^Δ166^) as compared with VAP^P58S^ (45.0±3.1 for VAP^Δ20^ and 46.6±0.06 for VAP^Δ166^). For each mutant, the value for wild type rescue differed significantly from mutant (*p*<0.05 for. Δ20 and 0.01 for Δ166; ANOVA with Sidak-Holm test). Rescue of lethality is also obtained by co-expressing VAP^P58S^ and VAP^wt^. In this case, the percentage of mutant males obtained was similar to that observed with VAP^P58S^ alone (45.7±3.3 for VAP^Δ20^ and 40.3±2.7 for VAP^Δ166^). However, for each mutant, rescue using the recombined VAP^P58S^, VAP^wt^ chromosome differed significantly from that obtained using VAP^wt^ alone (*p*<0.05 for Δ20 and 0.01 for Δ166, ANOVA with Sidak-Holm test). n.s., not significant. (B–E) VAP^P58S^ suppresses the thoracic bristle phenotype produced by expression of VAP^wt^ using *Sca*-GAL4. (B) Shown in this panel is the thorax of a control *(sca-*GAL4/+) adult fly with a stereotyped pattern and number of dorsal macrochaetae (arrows). (C) In *Sca*-GAL4/UAS-VAP^wt^ animals, the dorsal macrochaetae either fail to develop or are dramatically reduced in number. (D) Expression of VAP^P58S^ has no effect on the development or number of bristles. (E) However, co-expression of VAP^P58S^ with VAP^wt^ results in a suppression of the bristle loss phenotype caused due to overexpression of VAP^wt^.

The inability of VAP^P58S^ to rescue the bouton phenotype associated with VAP^Δ166^ shows that the ALS8-associated missense mutation impairs VAP function. This, together with the observation that neuronal expression of VAP^P58S^ increases bouton size similar to VAP loss of function, suggests that the mutation might function as a dominant negative. To explore this possibility, we compared the ability of VAP^wt^ and VAP^P58S^ to rescue the lethality associated with VAP loss of function. Since neuronal expression of VAP is sufficient to rescue lethality [12], we used *elav*-GAL4 to express VAP^wt^ and VAP^P58S^ transgenes in genetic backgrounds containing two independent VAP mutations: VAP^Δ20^ (a null allele) and VAP^Δ166^. Males hemizygous for these mutations do not survive to eclosion as adults. In both cases, the percentage of mutant VAP hemizygous males that eclosed as adults was higher with VAP^wt^ as compared to VAP^P58S^ (Figure 2A). The rescue by VAP^wt^ was approximately 63% and 33% greater as compared to VAP^P58S^ in VAP^Δ20^ and VAP^Δ166^ backgrounds, respectively. Of note, when VAP^wt^ and VAP^P58S^ were coexpressed, the percentage of rescued males that eclosed was very similar to that observed with VAP^P58S^ alone, suggesting that the ALS8-associated mutation may impair the function of the wild type protein, consistent with a dominant negative mode of action (Figure 2A).

We were intrigued by the apparent inhibition of normal neuronal VAP function by the ALS8 mutation and sought to examine the consequences of this putative dominant negative interaction in a different neuronal context. Overexpression of neurotoxic genes including mutant ataxin-1 in sensory precursors using the *scabrous*-GAL4 driver leads to loss of bristles in the adult [17]. We found that overexpression of VAP^wt^ using *scabrous*-GAL4 resulted in failure of thoracic macrochaetae to develop (Figure 2C). This bristle loss was not observed in *sca*-GAL4/UAS-VAP^P58S^ animals (Figure 2D). However, coexpression of VAP^P58S^ with VAP^wt^ suppressed the bristle phenotype associated with the latter (Figure 2E), again supporting a dominant negative effect of VAP^P58S^.

### VAP^P58S^ Appears to Aggregate and Recruit Normal VAP into Aggregates *In Vivo*

**Figure 3:**
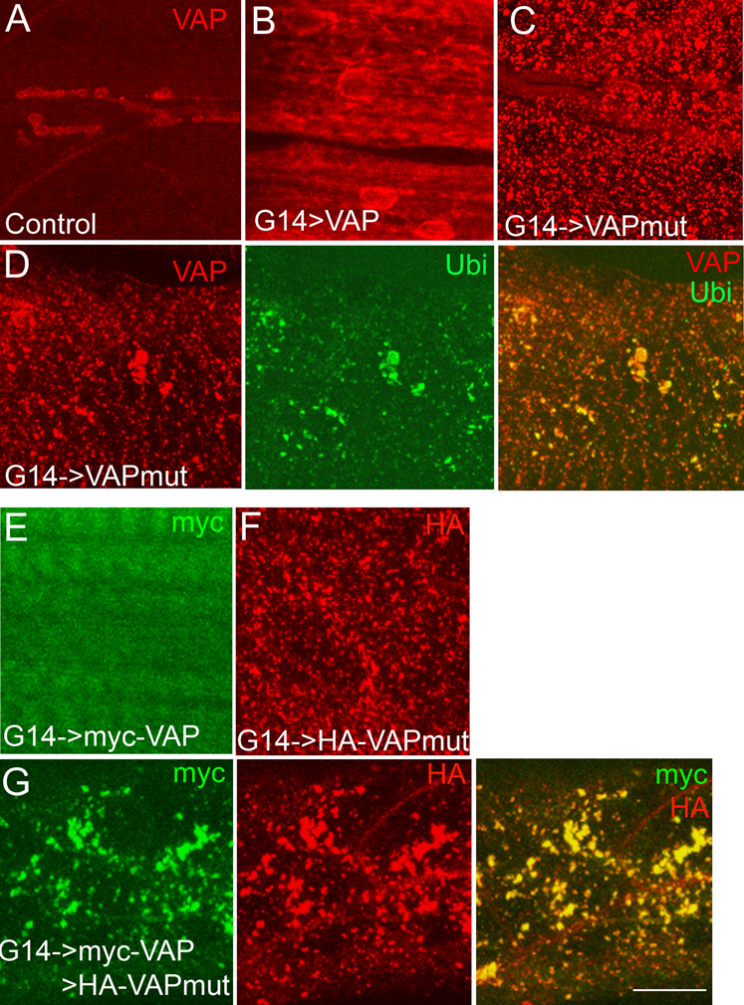
VAP^P58S^ forms ubiquitinated aggregates and induces aggregation of VAP^wt^ *in vivo*. (A–C) Confocal images of 3^rd^ instar larval muscles stained with anti-VAP. (A) A control animal (G14-GAL4/+). VAP expression is observed at the neuromuscular junction and in underlying muscles 6 and 7. (B) G14-GAL4/UAS-VAP^wt^. Increased VAP immunoreactivity is observed with expression of VAP^wt^. (C) G14-GAL4/UAS-VAP^P58S^. Punctate staining of ALS-associated mutant VAP is observed. (D) Staining with anti-VAP (red) and anti-ubiquitin (Ubi; green) of animals expressing VAP^P58S^ in the muscle. Mutant VAP forms aggregates that appeared as puncta through out the muscle (red). Ubiquitin immunoreactive puncta also are observed (green) that largely colocalized with the VAP aggregates (merged image). (E–G) Staining with anti-myc (green) and anti-HA (red) of animals expressing myc-VAP^wt^ and HA-VAP^P58S^ in the muscle. (E) Similar to untagged VAP^wt^ and VAP^P58S^ transgenes, expression of myc-VAP^wt^ appears diffuse and cytoplasmic. (F) HA-VAP^P58S^ forms aggregates that appear punctate throughout the muscle. (G) Muscle field of a 3^rd^ instar larva expressing both myc-VAP^wt^ and HA-VAP^P58S^. Expression of myc-VAP^wt^ (green) appears punctate and colocalizes with HA-VAP^P58S^ aggregates (merged image). Scale bar, 20 µm.

Abnormal aggregation of cellular proteins is a common feature of many neurodegenerative diseases, including Alzheimer's, Huntington's, and Parkinson's diseases, prion disorders, and ALS [18]–[22]. Biochemical studies have demonstrated that the ALS8 mutation results in insolubility and aggregate formation of VAPB in non-ER fractions [2], [13], [14]. To test if VAP^P58S^ formed aggregates similar to the human mutant VAPB protein, we compared the cellular distribution of wild type and ALS8 mutant VAP in the muscle of third instar larvae because of the ease with which this tissue can be visualized. Expression of VAP^wt^ resulted in a uniform increase in the intensity of VAP staining throughout the muscle (Figure 3B); on the other hand, expression of VAP^P58S^ resulted in a more punctate appearance in muscle (Figure 3C). To ensure that this apparent aggregate formation was independent of cellular context, we also examined the cellular distribution of VAP^wt^ and VAP^P58S^ in neurons of third instar larvae. As in muscle, neurons overexpressing VAP^P58S^ showed punctate cytoplasmic staining with reduced staining at the cell membrane, whereas more evenly distributed cytoplasmic staining was observed in neurons overexpressing VAP^wt^ (Figure S2).

Cellular deposits containing ubiquitin are characteristic of a number of neurodegenerative disorders, including ALS [23], [24]. We therefore examined ubiquitin staining in animals expressing VAP^wt^ and VAP^P58S^. In VAP^wt^ overexpressors, ubiquitin staining was similar to controls (data not shown). On the other hand, in animals overexpressing VAP^P58S^, punctate ubiquitin immunoreactivity was observed throughout the muscle. These puncta colocalized with VAP^P58S^ aggregates, suggesting that mutant VAP aggregates are ubiquitinated to be targeted for proteosomal degradation (Figure 3D).

One potential pathogenic mechanism underlying the ALS8 mutation would be recruitment of normal endogenous VAP into aggregates, thereby depleting the cell of wild type protein. To test this possibility, we generated transgenic flies carrying amino terminal hemagglutinin (HA) and Myc-tagged versions of VAP^P58S^ and VAP^wt^, respectively. These constructs were tested individually by expressing transgenes in muscle and staining with the appropriate epitope tag antibodies. Similar to the untagged form, Myc-VAP^wt^ staining was localized to the muscle membrane, whereas the HA-VAP^58S^ immunoreactivity appeared punctate throughout the muscle, suggesting that the tagged versions behave similar to the untagged forms (Figure 3E and F). However, when the two transgenes were expressed together, the Myc-VAP^wt^ staining appeared more punctate and largely colocalized with the HA immunoreactive puncta representing the ALS8 mutant VAP (Figure 3G). These data suggest that VAP^P58S^ may recruit the wild type protein into aggregates, consistent with a dominant negative effect whereby the normal protein thus recruited is functionally inhibited.

### VAP^P58S^ Impairs Microtubule Organization

**Figure 4:**
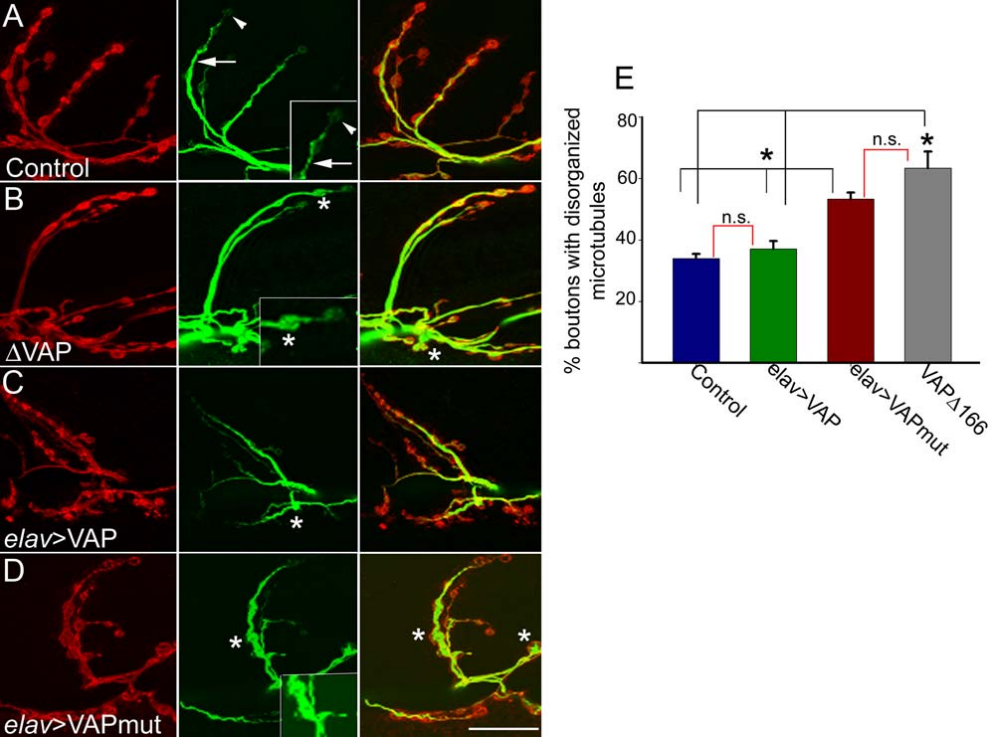
VAP^P58S^ disrupts microtubule organization. Representative synapses on muscle 6–7 of *elav*-GAL4/+ (A), VAP^Δ166^ (B), *elav*-GAL4/ UAS-VAP^wt^ (C) and *elav*-GAL4/UAS-VAP^P58S^ animals stained with anti-HRP to recognize the presynaptic membrane (red) and anti-Futsch (green). Anti-Futsch labels stable presynaptic microtubules. (A) In control animals, anti-Futsch staining appears thread-like (arrow). At distal boutons, the staining appears more punctate or diffuse (arrowhead). (B) In VAP^Δ166^ animals, Futsch expression fills up the cytoplasmic region inside boutons (asterisks). (C) In *elav*-GAL4/UAS-VAP^wt^ animals, the anti-Futsch staining appears similar to control animals, although some boutons show disorganized microtubule morphology (asterisk). (D) In *elav*-GAL4/UAS-VAP^P58S^, more boutons have a disorganized microtubule morphology (asterisk). (E) Quantitation of microtubule morphology assessed using anti-Futsch. The percentage of boutons of the muscle 6-7 synapse exhibiting disorganized microtubule phenotypes was calculated. Values: Controls: 34.0±1.5% (n = 26 synapses). *elav*-GAL4/ UAS-VAP^wt^: 37.0±2.6 % (n = 25 synapses). VAP^Δ166^ (63.4±5.4; n = 15 synapses). *elav*-GAL4/UAS-VAP^P58S^ (53.3±2.1 %; n = 40 synapses). The black brackets above the histogram indicate all significantly different relationships (*, *p*<0.001, ANOVA with Kruskal-Wallis test). The red brackets indicate non significant relationships. There was no significant difference in the percentage of boutons with abnormal microtubule morphology between VAP^Δ166^ and *elav*-GAL4/UAS-VAP^P58S^, nor was there a significant difference between elav-GAL4/UAS-VAP^wt^ and the control. Scale bar, 20 µm.

Impaired microtubule organization and microtubule dependent transport are believed to be a common underlying feature in many neurodegenerative diseases [25]. Microtubule-based transport plays a crucial role in survival of motor neurons in particular. Aberrant axonal transport contributes toward pathogenesis in sporadic ALS and in mice expressing mutant SOD1 [26]–[28]. Investigators previously have shown that VAP associates with microtubules [10], [12]. In the absence of VAP, microtubule organization at the neuromuscular junction is abnormal in *Drosophila* [12]. We were therefore interested in examining the effect of VAP^P58S^ on microtubule organization. We examined the neuromuscular junction in larvae expressing wild type and mutant VAP by immunohistochemistry using the monoclonal antibody 22C10, which recognizes Futsch, a presynaptic microtubule-associated protein related to vertebrate MAP1B [29]. The pattern of Futsch staining at the neuromuscular junction has been extensively characterized [12], [30]–[32]. In wild type larvae, Futsch staining (Figure 4A–D, green) in most boutons appears thread-like, running along the axis of each synaptic branch and continuous with the axonal cytoskeleton (Figure 4A, arrow; also see inset). At distal boutons, Futsch staining appears punctate or diffuse (Figure 4A, arrowhead; also see inset ). Occasionally, Futsch staining is found to fill the entire presynaptic space. These two forms of staining, i..e, “diffuse” and “filled”, have been described as two variant forms of disorganized microtubules found in mutants affecting synaptic architecture [30], [32]. In dVAP null mutants, approximately 70% of the boutons have been reported to show a filled (Figure 4B, asterisk) or diffuse pattern of Futsch immunoreactivity, consistent with reported microtubule disorganization [12]. To determine if expression of VAP^P58S^ also leads to disorganization of microtubules, we compared the pattern of Futsch staining at the neuromuscular junction of larvae expressing wild type and ALS8 mutant VAP in neurons. Similar to controls, in animals overexpressing VAP^wt^, the microtubules were well organized; 22C10 staining in most boutons was continuous, with few boutons displaying a diffuse or filled pattern of Futsch staining as compared to controls (Figure 4C and E; 33% in controls and 37% in VAP^wt^ overexpressors). In contrast, expression of VAP^P58S^ increased microtubule disorganization: 53% of boutons displayed either the filled or diffuse pattern of Futsch staining (Figure 4D and E). This finding was comparable to that observed in VAP^Δ166^ mutants, in which approximately 63% of the boutons showed disorganized microtubules (Figure 4E). Thus, microtubule organization is impaired by expression of ALS associated mutant VAP.

### Overexpression of VAP^wt^ But Not VAP^P58S^ Leads to a Decrease in the Number of Active Zones at the Neuromuscular Junction

**Figure 5:**
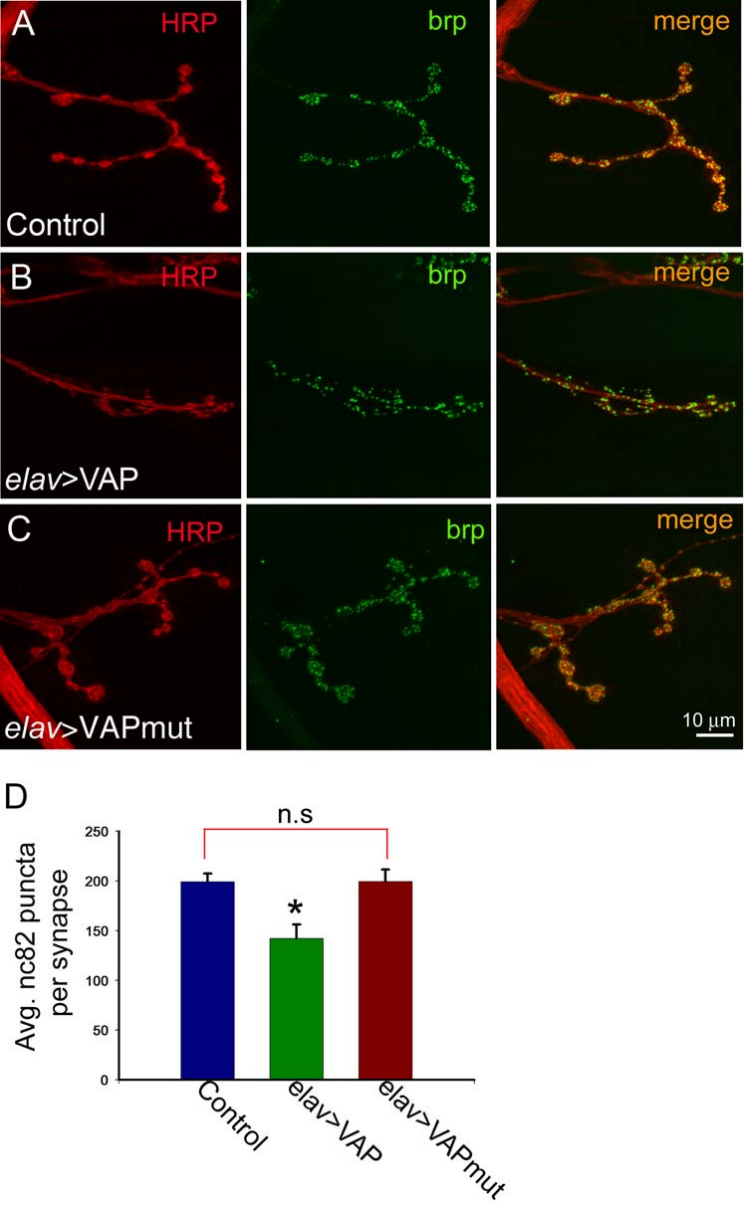
Neuronal overexpression of VAP^wt^ but not VAP^P58S^ leads a reduction in active zones. (A–C) Confocal images of synapses at muscle 4 stained with anti-HRP (red) and nc82, which labels the active zone protein bruchpilot (brp, green). Control (A) and *elav*-GAL4/UAS-VAP^P58S^ (C) animals showed similar numbers of nc82 immunoreactive puncta per synapse. In contrast, such puncta were fewer in *elav*-GAL4/UAS-VAP^wt^ animals (B). (D) A histogram showing the difference in number of nc82 positive puncta in all 3 genotypes. In *elav*-GAL4/UAS-VAP^wt^ animals, an average of 142±14 puncta per synapse were observed (n = 18) as compared to 199±8.4 (n = 17) and 199±12 (n = 16) in control and *elav*-GAL4/UAS-VAP^P58S^ animals, respectively (*, different from other two genotypes, *p*<0.001, ANOVA with Sidak-Holm test). n.s., not significant. Scale bar, 10 µm

Having established a putative dominant negative role for VAP^P58S^, we were interested in determining whether its expression in neurons altered the expression or localization of other synaptic proteins at the neuromuscular junction. Therefore, larvae expressing wild type and ALS8 mutant VAP were stained with antibodies against a variety of synaptic proteins such as synaptotagmin, syntaxin, and CSP. No obvious defects in the expression or localization of any of these markers were apparent (data not shown). However, some differences were observed when these animals were stained with nc82, a monoclonal antibody which recognizes Bruchpilot (*brp*), a crucial component found in active zones at the synapse [33], [34]. Despite an increase in the total number of boutons as a result of overexpression of VAP^wt^, there were fewer nc82 immunoreactive puncta per synapse in these animals (Figure 5B and D). Quantification of the total number of nc82 puncta per synapse at muscle 4 for each of the genotypes revealed a significant decrease in wild type overexpressors as compared to mutant and control larvae. No significant differences in the number of nc82 immunoreactive puncta were observed between controls and larvae expressing VAP^P58S^ (Figure 5C and D). To determine if the decrease in the total number of nc82 puncta was an indirect consequence of reduced bouton size caused by overexpression of VAP^wt^, we quantitated the number of the nc82 positive puncta relative to bouton cross sectional area in all three genotypes. In this case however, we did not observe any significant differences between the three genotypes (Table S1). This analysis suggests that the decrease in the total number of active zones per synapse might indeed be an effect of reduced bouton size rather than loss of active zones *per se*.

### Overexpression of VAP^wt^ and VAP^P58S^ Causes Ultrastructural Abnormalities at the Synapse

**Figure 6:**
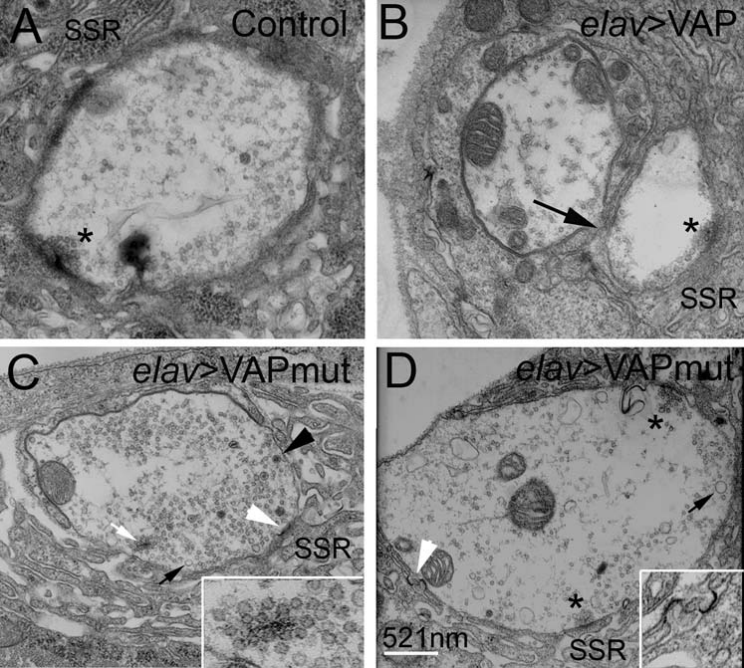
Ultrastructural analysis of the neuromuscular junction of animals overexpressing VAP^wt^ and VAP^P58S^. (A–D) Electron micrographs depicting cross sections of synaptic boutons in control (A), *elav*-GAL4/UAS-VAP^wt^ (B), and *elav*-GAL4/UAS-VAP^P58S^ larvae (C and D). Specialized membrane folds present on the postsynaptic surface called the subsynaptic reticulum (SSR), and active zones (asterisks) that include presynaptic T-bars are indicated. As compared to control boutons (A), boutons from animals overexpressing wild type VAP are smaller and contain fewer neurotransmitter vesicles (B). Occasionally, fusion of adjacent boutons was observed (arrow). (C and D) Boutons from *elav*-GAL4/VAP^P58S^ animals are shown. In addition to the smaller neurotransmitter containing vesicles, a few large vesicles were observed (C, dark arrow). Electron dense T-body-like structures associated with clusters of vesicles are often observed within the cytoplasm of the bouton (C, white arrow and inset). Abnormal electron dense structures are also found in the SSR close to the post-synaptic membrane (C and D, white arrowhead; inset in D).

Studies conducted in mutant SOD1 transgenic mouse models indicate that loss of synaptic connections, leading to nerve retraction, are early events in development of pathological phenotypes [35]–[37]. To evaluate whether expression of VAP^P58S^ causes structural abnormalities that might lead to synaptic instability, we conducted an ultrastructural analysis of the neuromuscular junction in VAP^wt^ and VAP^P58S^. At least five animals of each genotype were used for the analysis. The ultrastructure of a wild type bouton is shown in Figure 6A; notable structures include the subsynaptic reticulum (SSR), as well as the “T” bars associated with active zones (asterisk).

Overexpression of the wild type protein, as previously described (Figure 1), resulted in smaller boutons and, as previously reported by Pennetta and coworkers, these boutons also contained significantly fewer neurotransmitter vesicles [38] (Figure 6B). We observed a 54% decrease in the number of vesicles per bouton cross sectional area in these animals (*p*<0.001). In contrast, boutons from synapses overexpressing VAP^P58S^ contained abundant neurotransmitter vesicles, similar to controls (Figure 6C and D, see Table S2). A subset of these vesicles were abnormally large (Figure 6C and D, dark arrow) and sometimes filled with electron dense material (Figure 6C, dark arrowhead). We also occasionally observed abnormal electron dense structures in the SSR of these animals (Figure 6C and D, white arrowhead). We frequently observed electron dense structures attached to neurotransmitter vesicles floating within the cytoplasmic space of boutons and not associated with the presynaptic membrane (Figure 6D, white arrow). This phenotype, referred to as “floating T-bars” [39], was more frequently observed in animals expressing VAP^P58S^ (44% boutons in *elav*-GAL4/UAS-VAP^P58S^ versus 12.5 % in *elav*-GAL4/+ animals). In addition, there was a significant increase in the number of these floating T-bar structures in animals expressing VAP^P58S^ as compared to the controls (0.81±0.23 as expressed per bouton in *elav*-GAL4/UAS-VAP^P58S^ versus 0.25±0.17 in *elav*-GAL4/+ animals; *p*<0.05; see Table S3). We did not observe this phenotype in animals overexpressing VAP^wt^.

### Interaction between VAP and BMP signaling

The phenotype of floating T-bars that was sometimes observed in boutons of animals expressing VAP^P58S^ is reminiscent of the phenotype associated with mutations in components of the bone morphogenetic protein (BMP) signaling pathway [16], [39]–[41]. Given the resemblance between this phenotype and mutants of the BMP pathway, we sought to identify potential perturbations of BMP signaling in animals expressing the ALS8 mutation. In *Drosophila*, BMP signaling at the neuromuscular junction takes place in an anterograde as well as a retrograde manner and loss of BMP signaling leads to defects in synaptic growth and function [16], [39]–[41]. The signaling pathway involves activation of a heteromeric complex comprised of two type I and two type II receptor molecules. Upon ligand binding, the type II receptor phosphorylates the type I receptors, which transduce the signal by phosphorylating receptor-specific transcription factors called Smads. The phosphorylated Smad (pMAD in *Drosophila*) complex with Smad4 (Medea in *Drosophila*) translocates to the nucleus and activates transcription of target genes [42]. The level of activity of this pathway directly correlates with the level of phosphorylated Smad/pMAD; therefore, changes in levels of phosphorylated Smad or pMAD are used assess the activity of this signaling pathway [43], [44]. At the neuromuscular junction, pMAD accumulates at postsynaptic densities opposite the presynaptic active zone marker, bruchpilot (nc82) [45]. In motor neurons, pMAD accumulation takes place in response to retrograde signaling by the ligand *glass bottom boat* (*gbb*), mediated by the type II receptor *wishful thinking* (*wit*) [16].

**Figure 7:**
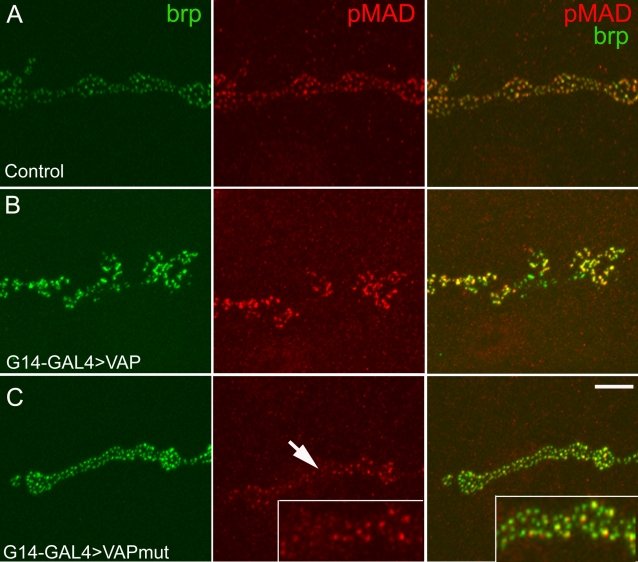
VAP^P58S^ impairs BMP signalling at the synapse. A–C, representative confocal images of the synapse at muscle 6–7 stained with phosphorylated SMAD (pMAD; red) and bruchpilot (nc82, green), a component of the active zone. (A) In control animals (G14-GAL4/+) postsynaptic expression of pMAD coincides with the expression of presynaptic Bruchpilot as shown in the merged image. (B) In G14-GAL4 /UAS-VAP^wt^ synapses, more intense pMAD staining is observed, indicative of enhanced BMP signalling. (C). In G14-GAL4/UAS-VAP^P58S^ animals, pMAD immunoreactive puncta are less intense (arrow) as compared to the controls indicating reduced BMP signaling. Scale bar, 10 µm.

In order to assess whether VAP^P58S^ affects BMP signaling, we expressed wild type and mutant transgenes in larval muscle using G14-GAL4 [16] and examined pMAD staining at the neuromuscular junction. As reported previously [45], in control animals, punctate pMAD staining was observed opposite presynaptic active zones immunostained with nc82 (Figure 7A). In VAP^wt^ transgenic animals, pMAD staining was more intense (Figure 7B) and a few nc82 immunoreactive puncta isolated from pMAD staining were also observed. In contrast, in animals expressing VAP^P58S^, the intensity of pMAD puncta was decreased (Figure 7C). We measured the intensity of pMAD puncta in all three genotypes. As compared to controls, in animals overexpressing VAP^wt^ we observed a 40% increase in intensity of pMAD staining, whereas expression of VAP^P58S^ resulted in a 42% decrease in intensity of pMAD staining. In animals expressing VAP^wt^, the average relative pixel intensity was 140.61±11.11 (mean±SEM). In VAP^P58S^ expressing animals, pixel intensity was 58.39±6.60 (mean±SEM). These values differed significantly (*p*<0.001 by ANOVA with Student-Neuman-Keuls test) from control pixel intensities, which were normalized to 100 (see Materials and Methods). We also examined pMAD expression in nuclei of motor neurons in the larval CNS. As at the neuromuscular junction, expression of VAP^P58S^ lead to a decrease in the intensity of pMAD staining (Figure S3), suggesting that both anterograde and retrograde BMP signaling may be impaired.

**Figure 8:**
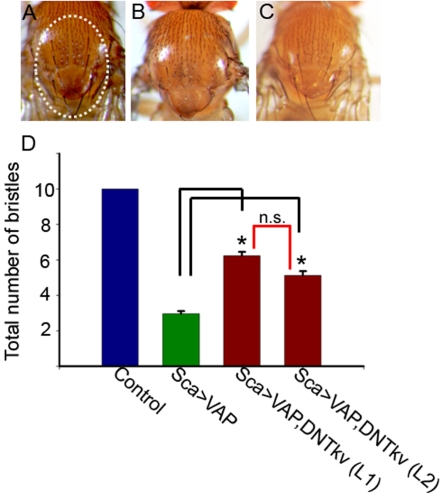
Genetic interaction between VAP and BMP signalling: Expression of dominant negative *thickvein* suppresses the VAP^wt^ bristle phenotype. (A–C) Thoraces of control (A), *sca*-GAL4/VAP^wt^ (B) and *sca*-GAL4/VAP^wt^, DNtkv animals (C). (A) In control animals, the bristles are invariant in number and position. Circled region indicates the 10 bristles used for analysis. (B and D) Expression of VAP^wt^ using *sca*-GAL4 leads to loss of bristles. On average, 2–3 bristles are observed in these animals. (C and D) Expression of dominant negative *thickvein* (DN-tkv) results in a significant suppression of the bristle loss phenotype caused by overexpression of VAP^wt^ (*p*<0.001, ANOVA with Sidak-Holm test). This was observed with two independent lines of DN-Tkv referred to as L1 and L2; an average of 6 and 5 bristles respectively, were observed in these animals (D). n.s., not significant.

These data indicate that increased VAP^wt^ expression potentiates BMP signaling, suggesting an interaction between VAP and BMP signaling pathways. In order to explore this possibility further, we conducted an epistasis analysis using the bristle phenotype observed in animals overexpressing VAP^wt^. Overexpression of VAP^wt^ using *sca*-GAL4 resulted in loss of thoracic bristles in adult flies (Figure 8B). To test if VAP and BMP signaling pathways interact, we co-expressed a dominant negative form of the type I BMP receptor-*thickvein* (DN-*tkv*). We found that co-expression of DN-*tkv* suppressed the bristle loss phenotype associated with overexpression of VAP^wt^ (Figure 8C). We quantitated this by counting the number of bristles present within a defined region of the thorax of adult flies. In control animals, the ten dorsal macrochaetae present in this region (circle in Figure 8A) were invariant in number and position in all adult flies examined (Figure 8D). However, in flies overexpressing VAP^wt^, an average of only 2–3 bristles were observed. Co-expression of DN-*tkv* using 2 independently derived transgenic lines resulted in significant suppression of the bristle loss phenotype: on average, 5–6 bristles were observed in these flies (Figure 8D). Taken together, these results are consistent with the assertion that VAP^P58S^ interferes with BMP signaling at the neuromuscular junction.

## Discussion

We have used *Drosophila* as a model system to gain insight into the mechanism by which the ALS8 associated mutation gives rise to disease. The evolutionary conservation of VAP and the availability of a vast array of genetic tools make the fly a particularly attractive model system with which to dissect pathophysiological mechanisms underlying ALS8. We have compared and assessed the cellular consequences of expression of wild type and ALS8-associated mutant VAP on the development and function of the synapse at the neuromuscular junction. In *Drosophila*, the synapses at the neuromuscular junction are glutamatergic, providing some similarities to the spinal cord synapse which are affected in ALS patients. Furthermore, the presynaptic neurons are obviously the functional equivalent to the lower motor neurons also affected in ALS patients.

The relationship between mutation and pathogenesis in inherited neurodegenerative disorders is obviously of great interest as it could point towards a mechanistic understanding of the disease and indicate treatment strategies. For genetically dominant disorders, potential mechanisms include toxic gain of function, haploinsufficiency, and dominant negative effects. Pennetta and coworkers concluded that the ALS8 mutation is a toxic gain of function rather than a dominant negative [38]. This conclusion was based on the toxic properties of the mutant protein in conjunction with its retention of activity. However, our data and that of others [13], [14] argue against retention of VAP activity by the ALS8 mutant, but rather suggest that that the ALS8 mutation in VAMP-associated-protein (VAP) functions through a dominant negative mechanism. Dominant negative mutations are those in which the product of the mutant gene adversely affects function of the normal gene product within the same cell, for example by forming non-functional dimers. Interestingly, members of the VAP family are able to form dimers [6], [8]. The following experiments reported here support the notion that the formation of mixed dimers or aggregates between wild type and ALS8 mutant proteins is at least in part responsible for the dominant phenotype. The VAP loss of function phenotype (VAP^Δ20^ and VAP^Δ166^) is characterized by an increase in bouton size and increased microtubule disorganization. Both these phenotypes are observed when VAP^P58S^ is overexpressed in neurons, indicating that expression of the mutant protein is dominant over the wild type protein. This is not simply due to an excess of mutant protein since VAP^P58S^ can also suppress the phenotypes generated by overexpression of VAP^wt^ (Figure 4). Finally, VAP^P58S^ forms ubiquitinated aggregates that can also recruit and induce aggregation of wild type protein (Figure 3), thereby rendering it functionally inactive. The ability of the mutant protein to recruit and induce aggregation of the wild type protein has also been reported for mammalian VAP *in vitro* [13], [14]. The finding that down regulation of mammalian VAP using RNAi techniques is sufficient to induce cell death in motor neurons [14] also argues that it is loss of VAP function, rather than the formation of aggregates, that is pathogenic.

VAP was originally identified as a “VAMP-associate protein” in *Aplysia*, and antibodies against VAP decrease synaptic transmission [10]. Subsequently, VAP was demonstrated to alter the size and number of boutons at the *Drosophila* neuromuscular junction [12]. Overexpression of VAP^wt^ leads to a significant decrease in the total number of active zone at the synapse despite an increase in bouton number; fewer nc82 immunoreactive puncta per synapse are present (Figure 5) even though the number of such puncta per unit cross sectional area of the bouton (µm^2^) is not significantly different from control and animals expressing mutant VAP. Further research will be necessary in order to determine whether this difference in the total number of active zones might lead to impaired synaptic transmission.

In a recent report, by conducting morophometric analysis on electron micrographs of individual boutons, Pennetta and colleagues show that there is no change in the number of active zones in animals overexpressing VAP^wt^ in neurons [38]. These authors also report locomotory defects, synaptic degeneration and muscle atrophy in larvae expressing VAP^P58S^ in neurons. We did not observe any of these defects in our experiments. Differences in expression levels of the mutant protein could account for these discrepancies.

Extensive crosstalk between motor neurons and muscle is a conspicuous feature of neuromuscular junction development in *Drosophila*, where synapses must accommodate the increase in size of muscles during larval development by increasing bouton number and active zones per bouton. Components of the TGF-β/ BMP signaling pathway are known to regulate neuronal survival, synaptic development and function in vertebrates as well as invertebrates. Recently, reduced TGF-β signaling has been associated with Alzheimers disease and the level of one of the receptors (TGFβRII) is reduced in brain tissue of Alzheimer's disease patients [46].

In *Drosophila*, retrograde BMP signaling is required for synaptic growth at the neuromuscular junction [39]. At the same time, anterograde BMP signaling in neurons appears to be essential to restore neurotransmission which is significantly lowered in mutants of the BMP pathway. Here, we show using epistasis analysis, the presence of a genetic interaction between VAP and BMP signaling pathways (Figure 8). We also show that overexpression of VAP^wt^ potentiates BMP signaling whereas overexpression of VAP^P58S^ impairs BMP signaling as evidenced by the decrease in intensity of pMAD puncta at the neuromuscular junction (Figure 7) and nuclei of motor neurons (Figure S3). Expression of VAP^P58S^ also causes ultrastructural phenotypes reminiscent of mutants in the BMP pathway, e.g., floating T-bars and occasional detached postsynaptic membranes. However, it should be noted that despite the similarities, these phenotypes are by no means identical; BMP mutants show smaller synapses (i.e., fewer boutons) with no significant change in bouton size- a phenotype unlike that observed in animals with neuronal expression of VAP^P58S^. This suggests that the aberrations associated with ALS8 are likely to be more complex than simply loss of receptors.

Recent observations that VAP is reduced in ALS patients and mutant SOD transgenic mice [14] suggest a potential role for VAP in many forms of ALS. Further genetic analysis using this model as a paradigm for ALS is likely to provide insights into pathways that contribute to motor neuron dysfunction and death in ALS.

## Materials and Methods

### *Drosophila* Genetics and Phenotypic Analysis

The VAP^Δ166^ and VAP^Δ20^ mutant lines and VAP cDNA were kindly provided by Hugo Bellen. G14-GAL4 and the second chromosome *elav*-GAL4 transgenic line were obtained from Kai Zinn. David Krantz provided the OK6-GAL4 line. The 24B-GAL4, tubulin-GAL4, actin-GAL4, *sca*-GAL4, and *repo*-GAL4 drivers were obtained from the Bloomington stock center. UAS-tkv^Δ^GSK (UAS-DNtkv) stocks were obtained from Michael O'Connor; both lines carried two insertions of the transgene [47].

Site-directed mutagenesis using the Stratagene Quick-Change II kit (Stratagene) was used to generate the P58S mutant using the wild type cDNA. This manipulation and placement of epitope tags were performed using the pGEM-7 cloning vector (Promega). Epitope tags (3X Myc or HA) were engineered at the amino terminus of wild type and P58S VAP using PCR. Identity of all constructs was verified by sequencing. Constructs were subcloned into the UAS expression vector and transgenic flies generated using standard techniques. Certain lines were generated using facilities of the Duke Model Systems Genomics Group. Multiple transgenic lines were generated and tested for each construct. At least two independently derived lines on the autosomes were tested for each construct. In no case were insertions selected on the basis of phenotypes or lack thereof. For all untagged experiments shown here, we used UAS-VAP^wt-A1.6^ and UAS-VAP^P58S-A2.1^, which showed similar *elav*-GAL4-driven expression levels as assessed using immunblots of head extracts in combination with guinea pig anti-VAP (Figure S1). For each construct, each of the lines used showed the same patterns of lethality under control of various drivers. Neuronal overexpression with *elav*-GAL4 was carried out at 30°C. Experiments involving overexpression in the muscle were conducted at 25°C. Bristle phenotype and all rescue experiments were carried out at 23°C.

We generated animals with both HA and myc tags for both the ALS8 and wild type transgenes in order to exclude the possibility that aggregation was due to the epitope tag alone (data not shown). For co-expression studies, mitotic recombination was used to place both UAS-VAP^wt^ and UAS-VAP^P58S^ transgenes on the second chromosome. For studies of transgene expression in genetic backgrounds lacking endogenous VAP, VAP^Δ166^ /FM7Kr-GFP; *elav*-GAL4 females were crossed to w/Y; UAS-VAP males (either wild type, P58S, or the recombined chromosome containing both transgenes).

### Immunohistochemistry

Wandering third instar larvae were dissected in PBS and fixed in Bouin's fixative (picric acid+37% formaldehyde+glacial acetic acid, 15:5:1) for 10 min for staining using guinea pig anti-VAP and 20 min for all other antibodies. The only exception for fixative was for experiments using pMAD, in which 4% paraformaldehyde in PBS was used. The synapse on muscle 4 was used to carry out bouton size measurement. This muscle receives innervations from 3 motor neurons [48]. However, we examined only the size of type1b boutons, which show robust Dlg staining. In our experiments, only synapses present in segments A2 and A3 were examined [31]. Except as noted, all antibodies were murine monoclonals obtained from the Developmental Studies Hybridoma Bank. Primary antibodies used were anti-HRP (Sigma; 1∶500), 22C10 (anti-Futsch; 1∶50), NC82 (anti-bruchpilot; 1∶50), anti-ubiquitin (FK2, Biomol, 1∶500), anti-Myc (9E10; 1∶50), chicken anti-HA (Aves; 1∶500), anti-pMAD (1:500,), rabbit anti-Dlg (1:40,000), 4F3 ( anti-Dlg; 1∶50), 8C3 (anti-Syntaxin; 1∶25), and guinea pig anti-VAP (1∶200). Anti-VAP was obtained from Hugo Bellen, Dlg from Vivian Budnik, and pMad from Peter ten Dijke [49]. Staining was visualized using Alexafluor 488 and 568 anti-mouse, -rabbit, -guinea-pig, or chick secondary antibodies (Invitrogen; 1∶500).

### Immunoblotting

Immunoblot analysis of adult heads was carried out using published methods [50] in conjunction with guinea pig anti-VAP (1∶10,000) and mouse anti-β-tubulin (1∶1000; Accurate Chemical and Scientific).

### Image Acquisition and Processing

All confocal images were obtained using a Zeiss Pascal laser scanning confocal microscope in conjunction with a 63X NA 1.4 Zeiss Apochromat or 40X NA 0.75 Axoplan Neofluar lens at room temperature using Vectashield (Vector labs) as an imaging medium and the Zeiss Pascal software. For experiments in which direct comparisons were made based on fluorescence intensity (i.e., Figure 7 and Figure S3) images for different genotypes were acquired under precisely identical conditions and in succession. Images were processed in Adobe Photoshop. Films from immunoblots were scanned with a UMAX Powerlook 1000 transmissive flatbed scanner. Graphical and statistical analyses were performed using SigmaPlot 9.0 and Systat 3.1.

For quantitation of the relative fluorescence intensity levels of pMAD, the pixel intensity of pMAD puncta for all three genotypes (control, G14-GAL4>VAP^wt^ and G14-GAL4>VAP^P58S^) was measured using Image J software. Three independent sets of experiments were conducted, and in each case all three genotypes were imaged simultaneously using identical imaging conditions. The change in intensity stated in the text represents the average change observed in these three independent experiments. For each set, 5–8 synapses of each genotype were imaged. The percentage relative fluorescence intensity of in G14-GAL4>VAP^wt^ and G14-GAL4>VAP^P58S^ was calculated by normalizing the control intensity to 100 percent.

### Electron Microscopy

Third instar larvae were filleted and pinned first in normal saline, then glutaraldehyde (2% in 0.12 M sodium cacodylate buffer, pH 7.4) for 2 hr with rotation. Samples then were rinsed thrice in 0.12 M sodium cacodylate buffer and postfixed with 1% osmium tetroxide in the same buffer with rotation. After postfixation, samples were washed and stained with 1% aqueous uranyl acetate for 1 hr. Specimens then were washed, dehydrated, and embedded in Eponate 12 resin. Thin sections were stained with uranyl acetate and Sato's lead. Analysis was carried out using a JEOL 100CX transmission electron microscope at an operating voltage of 80 kV.

## Supporting Information

Figure S1 Comparable expression of UAS-VAP^wt-A1.6^ and UAS-VAP^P58S-A2.1^. Immunoblot using head extracts from *elav*-GAL4>VAP^wt^ and *elav*-GAL4>VAP^P58S^ animals. The same blot was stripped and reprobed with anti-β-tubulin.
(0.69 MB TIF)

Figure S2 A comparison of the cellular staining pattern of VAP^wt^ and VAP^P58S^ in neurons. Shown are images of the 3rd instar larval brain from *elav*-GAL4/VAP^wt^ (A) and *elav*-GAL4/VAP^P58S^ animals (B). Green, anti-HRP staining; red, anti-VAP. Overexpression of VAP^wt^ leads to robust intracellular staining (A). In contrast, expression of VAP^P58S^ leads to formation of aggregates (B). Scale bar, 10 µm.
(1.86 MB TIF)

Figure S3 Muscle expression of VAP^wt^ causes an increase in pMAD accumulation in neuronal nuclei of the larval CNS. (A–C) Representative images of the CNS of 3rd instar larvae stained with an antibody against phophorylated MAD (pMAD) protein. (A) Control (G14-GAL4/+) animal showing the nuclear accumulation of phosphorylated MAD. (B) CNS of G14-GAL4/ UAS-VAP^wt^ animal. An increase in pMAD staining is observed. (C) CNS of G14-GAL4/UAS-VAP^P58S^ animal. A decrease in pMAD staining is observed. Scale bar, 10 µm.
(1.13 MB TIF)

Table S1 Analysis of the number of the nc82 immunoreactive puncta relative to bouton cross sectional area. Values shown are mean±SEM, N = 8 for each genotype; one-way Kruskal-Wallis ANOVA. There were no significant differences observed, suggesting that the apparent decrease in the total number of active zones per synapse might indeed be an effect of reduced bouton size rather than loss of active zones.
(0.04 MB DOC)

Table S2 Quantitation of neurotransmitter vesicle number per bouton cross sectional area. Values shown are mean±SEM, N = 9 for Driver alone and VAPwt and 10 for VAPmut; one-way ANOVA with Student-Newman-Keuls comparison; each bouton analyzed was considered as an independent sample for this analysis irrespective of the animal from which it was obtained. Neuronal expression of wild type VAP significantly reduced vesicle numbers as compared to the driver alone or mutant VAP.
(0.04 MB DOC)

Table S3 Quantitation of floating T bars per bouton. Values indicated are mean±SEM, N = 16 for Driver alone, 17 for VAPwt, and 27 for VAPmut; one-way ANOVA with Student-Newman-Keuls comparison; each bouton analyzed was considered as an independent sample for this analysis irrespective of the animal from which it was obtained. Neuronal expression of mutant VAP significantly increased the number of floating T bars as compared to the driver alone or mutant VAP.
(0.04 MB DOC)

## References

[1] Cleveland DW, Rothstein JD (2001). From Charcot to Lou Gehrig: deciphering selective motor neuron death in ALS.. Nat Rev Neurosci.

[2] Nishimura AL, Mitne-Neto M, Silva HC, Richieri-Costa A, Middleton S (2004). A mutation in the vesicle-trafficking protein VAPB causes late-onset spinal muscular atrophy and amyotrophic lateral sclerosis.. Am J Hum Genet.

[3] Marques VD, Barreira AA, Davis MB, Abou-Sleiman PM, Silva WA (2006). Expanding the phenotypes of the Pro56Ser VAPB mutation: proximal SMA with dysautonomia.. Muscle Nerve.

[4] Nishimura Y, Hayashi M, Inada H, Tanaka T (1999). Molecular cloning and characterization of mammalian homologues of vesicle-associated membrane protein-associated (VAMP-associated) proteins.. Biochem Biophys Res Commun.

[5] Weir ML, Klip A, Trimble WS (1998). Identification of a human homologue of the vesicle-associated membrane protein (VAMP)-associated protein of 33 kDa (VAP-33): a broadly expressed protein that binds to VAMP.. Biochem J.

[6] Kaiser SE, Brickner JH, Reilein AR, Fenn TD, Walter P (2005). Structural basis of FFAT motif-mediated ER targeting.. Structure.

[7] Italiano JE, Stewart M, Roberts TM (2001). How the assembly dynamics of the nematode major sperm protein generate amoeboid cell motility.. Int Rev Cytol.

[8] Amarilio R, Ramachandran S, Sabanay H, Lev S (2005). Differential regulation of endoplasmic reticulum structure through VAP-Nir protein interaction.. J Biol Chem.

[9] Foster LJ, Weir ML, Lim DY, Liu Z, Trimble WS (2000). A functional role for VAP-33 in insulin-stimulated GLUT4 traffic.. Traffic.

[10] Skehel PA, Martin KC, Kandel ER, Bartsch D (1995). A VAMP-binding protein from Aplysia required for neurotransmitter release.. Science.

[11] Kagiwada S, Hosaka K, Murata M, Nikawa J, Takatsuki A (1998). The Saccharomyces cerevisiae SCS2 gene product, a homolog of a synaptobrevin-associated protein, is an integral membrane protein of the endoplasmic reticulum and is required for inositol metabolism.. J Bacteriol.

[12] Pennetta G, Hiesinger PR, Fabian-Fine R, Meinertzhagen IA, Bellen HJ (2002). Drosophila VAP-33A directs bouton formation at neuromuscular junctions in a dosage-dependent manner.. Neuron.

[13] Kanekura K, Nishimoto I, Aiso S, Matsuoka M (2006). Characterization of amyotrophic lateral sclerosis-linked P56S mutation of vesicle-associated membrane protein-associated protein B (VAPB/ALS8).. J Biol Chem.

[14] Teuling E, Ahmed S, Haasdijk E, Demmers J, Steinmetz MO (2007). Motor neuron disease-associated mutant vesicle-associated membrane protein-associated protein (VAP) B recruits wild-type VAPs into endoplasmic reticulum-derived tubular aggregates.. J Neurosci.

[15] Brand AH, Perrimon N (1993). Targeted gene expression as a means of altering cell fates and generating dominant phenotypes.. Development.

[16] Aberle H, Haghighi AP, Fetter RD, McCabe BD, Magalhaes TR (2002). wishful thinking encodes a BMP type II receptor that regulates synaptic growth in Drosophila.. Neuron.

[17] Tsuda H, Jafar-Nejad H, Patel AJ, Sun Y, Chen HK (2005). The AXH domain of Ataxin-1 mediates neurodegeneration through its interaction with Gfi-1/Senseless proteins.. Cell.

[18] Haass C, Selkoe DJ (2007). Soluble protein oligomers in neurodegeneration: lessons from the Alzheimer's amyloid beta-peptide.. Nat Rev Mol Cell Biol.

[19] Bruijn LI, Houseweart MK, Kato S, Anderson KL, Anderson SD (1998). Aggregation and motor neuron toxicity of an ALS-linked SOD1 mutant independent from wild-type SOD1.. Science.

[20] Forman MS, Trojanowski JQ, Lee VM (2004). Neurodegenerative diseases: a decade of discoveries paves the way for therapeutic breakthroughs.. Nat Med.

[21] Soto C, Estrada L, Castilla J (2006). Amyloids, prions and the inherent infectious nature of misfolded protein aggregates.. Trends Biochem Sci.

[22] Orr HT, Zoghbi HY (2007). Trinucleotide repeat disorders.. Annu Rev Neurosci.

[23] Ciechanover A, Brundin P (2003). The ubiquitin proteasome system in neurodegenerative diseases: sometimes the chicken, sometimes the egg.. Neuron.

[24] Ross CA, Pickart CM (2004). The ubiquitin-proteasome pathway in Parkinson's disease and other neurodegenerative diseases.. Trends Cell Biol.

[25] Cairns NJ, Lee VM, Trojanowski JQ (2004). The cytoskeleton in neurodegenerative diseases.. J Pathol.

[26] Collard JF, Cote F, Julien JP (1995). Defective axonal transport in a transgenic mouse model of amyotrophic lateral sclerosis.. Nature.

[27] Sasaki S, Iwata M (1996). Impairment of fast axonal transport in the proximal axons of anterior horn neurons in amyotrophic lateral sclerosis.. Neurology.

[28] Williamson TL, Cleveland DW (1999). Slowing of axonal transport is a very early event in the toxicity of ALS-linked SOD1 mutants to motor neurons.. Nat Neurosci.

[29] Hummel T, Krukkert K, Roos J, Davis G, Klambt C (2000). Drosophila Futsch/22C10 is a MAP1B-like protein required for dendritic and axonal development.. Neuron.

[30] Roos J, Hummel T, Ng N, Klambt C, Davis GW (2000). Drosophila Futsch regulates synaptic microtubule organization and is necessary for synaptic growth.. Neuron.

[31] Sherwood NT, Sun Q, Xue M, Zhang B, Zinn K (2004). Drosophila spastin regulates synaptic microtubule networks and is required for normal motor function.. PLoS Biol.

[32] Packard M, Koo ES, Gorczyca M, Sharpe J, Cumberledge S (2002). The Drosophila Wnt, wingless, provides an essential signal for pre- and postsynaptic differentiation.. Cell.

[33] Wagh DA, Rasse TM, Asan E, Hofbauer A, Schwenkert I (2006). Bruchpilot, a protein with homology to ELKS/CAST, is required for structural integrity and function of synaptic active zones in Drosophila.. Neuron.

[34] Kittel RJ, Wichmann C, Rasse TM, Fouquet W, Schmidt M (2006). Bruchpilot promotes active zone assembly, Ca2+ channel clustering, and vesicle release.. Science.

[35] Fischer LR, Culver DG, Tennant P, Davis AA, Wang M (2004). Amyotrophic lateral sclerosis is a distal axonopathy: evidence in mice and man.. Exp Neurol.

[36] Frey D, Schneider C, Xu L, Borg J, Spooren W (2000). Early and selective loss of neuromuscular synapse subtypes with low sprouting competence in motoneuron diseases.. J Neurosci.

[37] Pun S, Santos AF, Saxena S, Xu L, Caroni P (2006). Selective vulnerability and pruning of phasic motoneuron axons in motoneuron disease alleviated by CNTF.. Nat Neurosci.

[38] Chai A, Withers J, Koh YH, Parry K, Bao H (2008). hVAPB, the causative gene of a heterogeneous group of motor neuron diseases in humans, is functionally interchangeable with its Drosophila homologue DVAP-33A at the neuromuscular junction.. Hum Mol Genet.

[39] McCabe BD, Marques G, Haghighi AP, Fetter RD, Crotty ML (2003). The BMP homolog Gbb provides a retrograde signal that regulates synaptic growth at the Drosophila neuromuscular junction.. Neuron.

[40] McCabe BD, Hom S, Aberle H, Fetter RD, Marques G (2004). Highwire regulates presynaptic BMP signaling essential for synaptic growth.. Neuron.

[41] Marques G, Haerry TE, Crotty ML, Xue M, Zhang B (2003). Retrograde Gbb signaling through the Bmp type 2 receptor wishful thinking regulates systemic FMRFa expression in Drosophila.. Development.

[42] Massague J (1996). TGFbeta signaling: receptors, transducers, and Mad proteins.. Cell.

[43] Shimizu K, Gurdon JB (1999). A quantitative analysis of signal transduction from activin receptor to nucleus and its relevance to morphogen gradient interpretation.. Proc Natl Acad Sci U S A.

[44] Tanimoto H, Itoh S, ten Dijke P, Tabata T (2000). Hedgehog creates a gradient of DPP activity in Drosophila wing imaginal discs.. Mol Cell.

[45] Dudu V, Bittig T, Entchev E, Kicheva A, Julicher F (2006). Postsynaptic mad signaling at the Drosophila neuromuscular junction.. Curr Biol.

[46] Tesseur I, Zou K, Esposito L, Bard F, Berber E (2006). Deficiency in neuronal TGF-beta signaling promotes neurodegeneration and Alzheimer's pathology.. J Clin Invest.

[47] Haerry TE, Khalsa O, O'Connor MB, Wharton KA (1998). Synergistic signaling by two BMP ligands through the SAX and TKV receptors controls wing growth and patterning in Drosophila.. Development.

[48] Hoang B, Chiba A (2001). Single-cell analysis of Drosophila larval neuromuscular synapses.. Dev Biol.

[49] Persson U, Izumi H, Souchelnytskyi S, Itoh S, Grimsby S (1998). The L45 loop in type I receptors for TGF-beta family members is a critical determinant in specifying Smad isoform activation.. FEBS Lett.

[50] Sang TK, Chang HY, Lawless GM, Ratnaparkhi A, Mee L (2007). A Drosophila model of mutant human parkin-induced toxicity demonstrates selective loss of dopaminergic neurons and dependence on cellular dopamine.. J Neurosci.

