# Supplementary figures and images for: A Drosophila Model of ALS: Human ALS-Associated Mutation in VAP33A Suggests a Dominant Negative Mechanism
Source: PLoS One. 2008 Jun 4;3(6):e2334. doi: 10.1371/journal.pone.0002334 (PMC2390852; doi:10.1371/journal.pone.0002334)

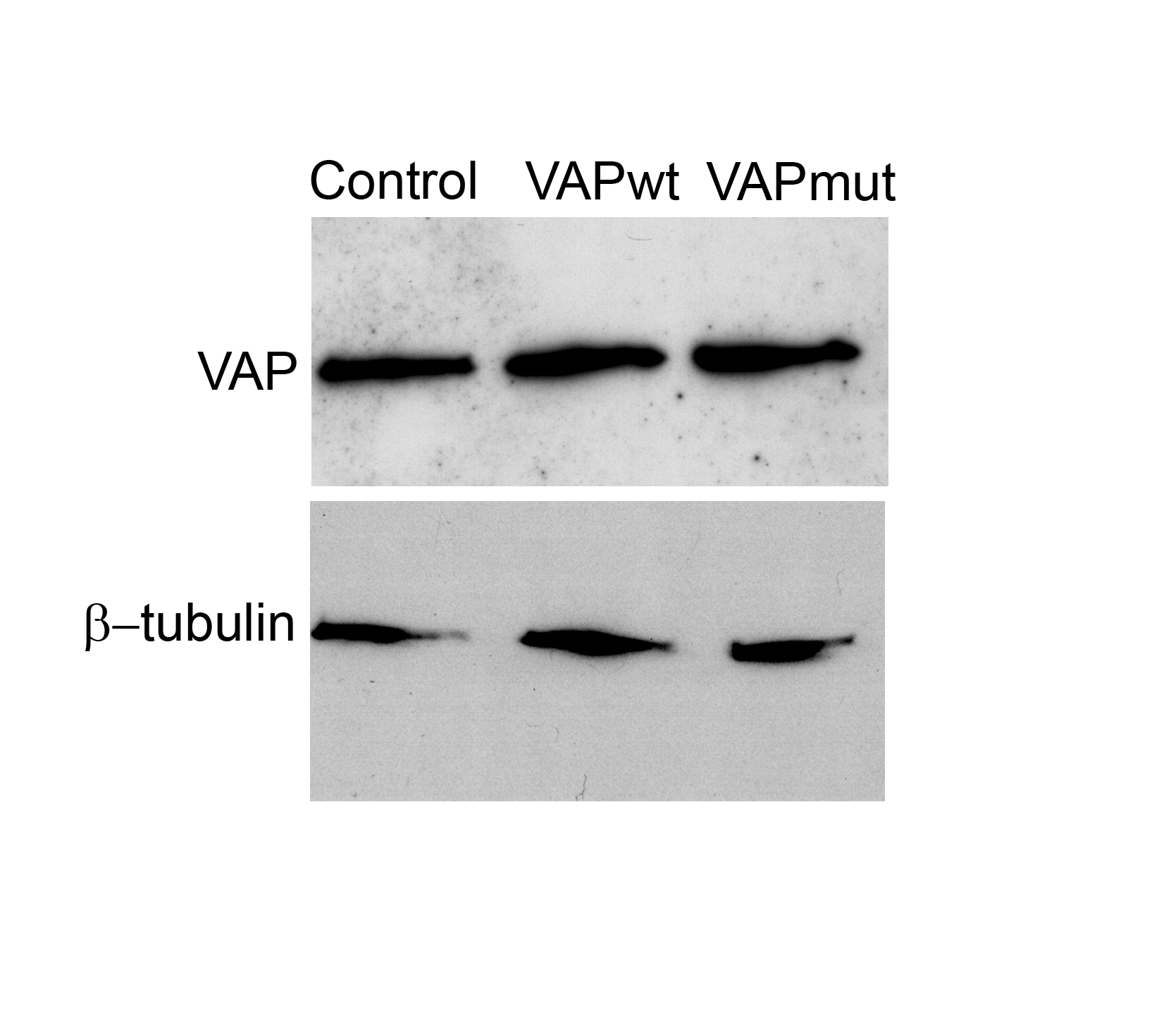

Supplement: Figure S1 — Comparable expression of UAS-VAPwt-A1.6 and UAS-VAPP58S-A2.1. Immunoblot using head extracts from elav-GAL4>VAPwt and elav-GAL4>VAPP58S animals. The same blot was stripped and reprobed with anti-β-tubulin. (0.69 MB TIF) [file pone.0002334.s001.tif]

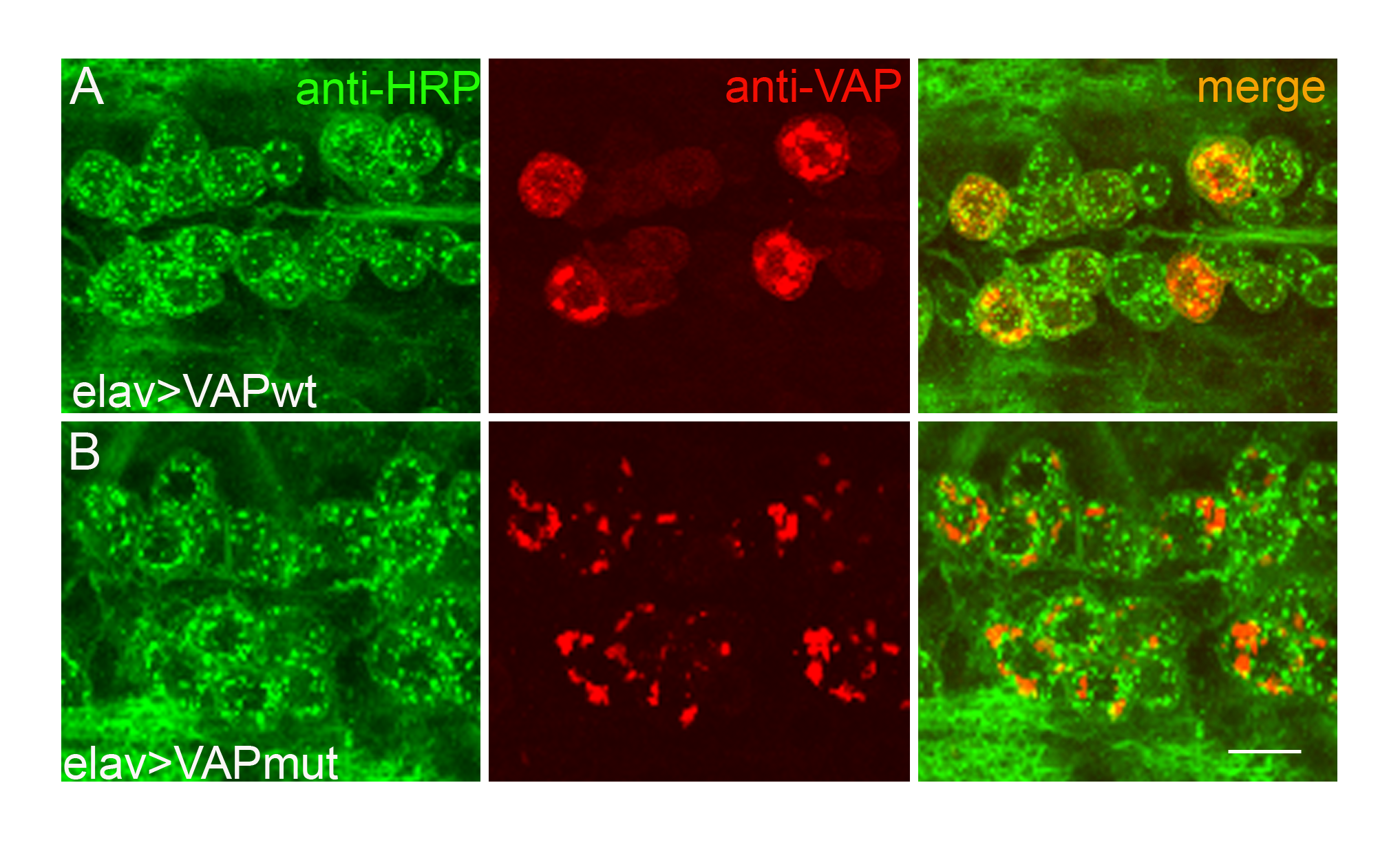

Supplement: Figure S2 — A comparison of the cellular staining pattern of VAPwt and VAPP58S in neurons. Shown are images of the 3rd instar larval brain from elav-GAL4/VAPwt (A) and elav-GAL4/VAPP58S animals (B). Green, anti-HRP staining; red, anti-VAP. Overexpression of VAPwt leads to robust intracellular staining (A). In contrast, expression of VAPP58S leads to formation of aggregates (B). Scale bar, 10 µm. (1.86 MB TIF) [file pone.0002334.s002.tif]

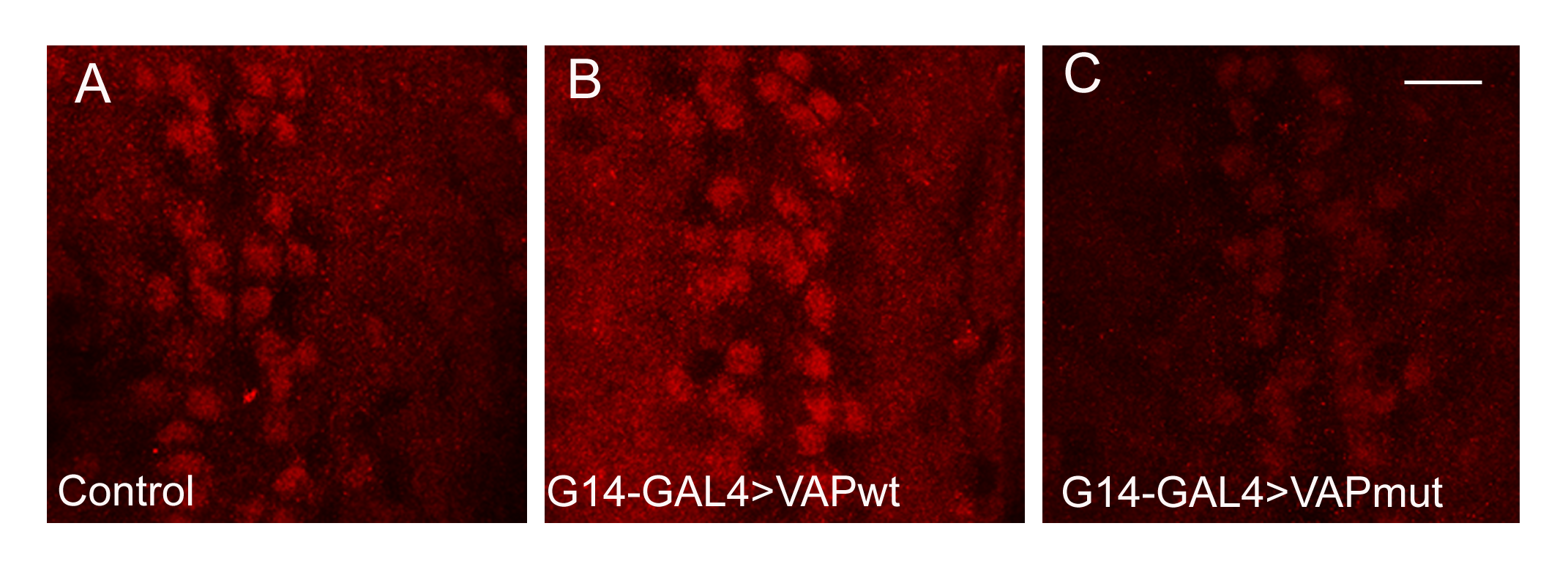

Supplement: Figure S3 — Muscle expression of VAPwt causes an increase in pMAD accumulation in neuronal nuclei of the larval CNS. (A–C) Representative images of the CNS of 3rd instar larvae stained with an antibody against phophorylated MAD (pMAD) protein. (A) Control (G14-GAL4/+) animal showing the nuclear accumulation of phosphorylated MAD. (B) CNS of G14-GAL4/ UAS-VAPwt animal. An increase in pMAD staining is observed. (C) CNS of G14-GAL4/UAS-VAPP58S animal. A decrease in pMAD staining is observed. Scale bar, 10 µm. (1.13 MB TIF) [file pone.0002334.s003.tif]
