# Supplementary material for: A Drosophila Model of ALS: Human ALS-Associated Mutation in VAP33A Suggests a Dominant Negative Mechanism
Source: PLoS One. 2008 Jun 4;3(6):e2334. doi: 10.1371/journal.pone.0002334 (PMC2390852; doi:10.1371/journal.pone.0002334)
Supplement: Table S1 — Analysis of the number of the nc82 immunoreactive puncta relative to bouton cross sectional area. Values shown are mean±SEM, N = 8 for each genotype; one-way Kruskal-Wallis ANOVA. There were no significant differences observed, suggesting that the apparent decrease in the total number of active zones per synapse might indeed be an effect of reduced bouton size rather than loss of active zones. (0.04 MB DOC) [file pone.0002334.s004.doc]

|  |  | vs. Driver | vs. VAPwt | Vs. VAPmut |
| --- | --- | --- | --- | --- |
| Driver | 1.58 + 0.11 |  | NS | NS |
| VAPwt | 1.71 + 0.14 | NS |  | NS |
| VAPmut | 1.32 + 0.04 | NS | NS |  |
