# Supplementary material for: A Drosophila Model of ALS: Human ALS-Associated Mutation in VAP33A Suggests a Dominant Negative Mechanism
Source: PLoS One. 2008 Jun 4;3(6):e2334. doi: 10.1371/journal.pone.0002334 (PMC2390852; doi:10.1371/journal.pone.0002334)
Supplement: Table S2 — Quantitation of neurotransmitter vesicle number per bouton cross sectional area. Values shown are mean±SEM, N = 9 for Driver alone and VAPwt and 10 for VAPmut; one-way ANOVA with Student-Newman-Keuls comparison; each bouton analyzed was considered as an independent sample for this analysis irrespective of the animal from which it was obtained. Neuronal expression of wild type VAP significantly reduced vesicle numbers as compared to the driver alone or mutant VAP. (0.04 MB DOC) [file pone.0002334.s005.doc]

|  |  | vs. Driver | vs. VAPwt | Vs. VAPmut |
| --- | --- | --- | --- | --- |
| Driver | 111.7 + 10.8 |  | *p* < 0.01 | NS |
| VAPwt | 60.3 + 11.3 | *p* < 0.01 |  | *p* < 0.001 |
| VAPmut | 136.0 + 14.2 | NS | *p* < 0.001 |  |
