# Supplementary material for: A Drosophila Model of ALS: Human ALS-Associated Mutation in VAP33A Suggests a Dominant Negative Mechanism
Source: PLoS One. 2008 Jun 4;3(6):e2334. doi: 10.1371/journal.pone.0002334 (PMC2390852; doi:10.1371/journal.pone.0002334)
Supplement: Table S3 — Quantitation of floating T bars per bouton. Values indicated are mean±SEM, N = 16 for Driver alone, 17 for VAPwt, and 27 for VAPmut; one-way ANOVA with Student-Newman-Keuls comparison; each bouton analyzed was considered as an independent sample for this analysis irrespective of the animal from which it was obtained. Neuronal expression of mutant VAP significantly increased the number of floating T bars as compared to the driver alone or mutant VAP. (0.04 MB DOC) [file pone.0002334.s006.doc]

|  |  | vs. Driver | vs. VAPwt | Vs. VAPmut |
| --- | --- | --- | --- | --- |
| Driver | 0.25 + 0.17 |  | NS | *p* < 0.05 |
| VAPwt | 0 + 0 | NS |  | *p* < 0.05 |
| VAPmut | 0.81 + 0.23 | *p* < 0.05 | *p* < 0.05 |  |
